# A COVID‐19 human viral challenge model. Learning from experience

**DOI:** 10.1111/irv.12797

**Published:** 2020-08-12

**Authors:** Rob Lambkin‐Williams, John P. DeVincenzo

**Affiliations:** ^1^ VirologyConsult Ltd. Brighton UK; ^2^ University of Tennessee School of Medicine Memphis TN USA

**Keywords:** controlled human infection model, COVID‐19, human viral challenge model, RSV, SARS‐CoV‐19

## Abstract

The controlled human infection model and specifically the human viral challenge model are not dissimilar to standard clinical trials while adding another layer of complexity and safety considerations. The models deliberately infect volunteers, with an infectious challenge agent to determine the effect of the infection and the potential benefits of the experimental interventions. The human viral challenge model studies can shorten the time to assess the efficacy of a new vaccine or treatment by combining this with the assessment of safety. The newly emerging SARS‐CoV‐2 virus is highly contagious, and an urgent race is on to develop a new vaccine against this virus in a timeframe never attempted before. The use of the human viral challenge model has been proposed to accelerate the development of the vaccine. In the early 2000s, the authors successfully developed a pathogenic human viral challenge model for another virus for which there was no effective treatment and established it to evaluate potential therapies and vaccines against respiratory syncytial virus. Experience gained in the development of that model can help with the development of a COVID‐19 HVCM and the authors describe it here.

## 1 INTRODUCTION

The world is now faced with a significant public health crisis. The unique features of SARS‐CoV‐2^1^ human infections, mainly its relatively long pre‐symptomatic but infectious phase has meant that traditional methods of quarantine and “social distancing” are less effective than anticipated. On 11 February, the disease caused by SARS‐CoV‐2 was named COVID‐19 by the World Health Organization (WHO).^2^

Although SARS‐CoV‐2 virus can be transmitted by aerosols,^3^ it is believed to be transmitted primarily by respiratory droplets and contact routes.^4^ The virus also has a high R_0_ (basic reproductive number),^5^ when combined with the extended period of pre‐symptomatic prolonged shedding of infectious virus,^6^ and complete lack of pre‐existing immunity in the world's population meant the virus spread rapidly around the world and was declared a pandemic by the World Health Organization on the 11 March 2020.^2^

This virus has continued to spread despite even draconian social distancing, which once relaxed, may still leave much of the world without immunity after this first wave.^7^ Moreover, the economic and social negative impact of such measures cannot be withstood for the long durations of the predicted global viral pandemic circulation.^8^ Consequently, there is a tremendous need for effective vaccines, mAbs, immunomodulators, antivirals and therapeutic interventions which must be developed quickly, efficiently, and with high scientific and ethical rigour.

Historically, controlled human infection studies (CHIMS) and specifically human viral challenge model (HVCM) have rapidly advanced the development of interventions for many infectious diseases. Recognising their potential advantages for controlling the COVID‐19 pandemic, the World Health Organization has recently issued guidelines on such studies.^9^ A COVID‐19 HVCM would be an important tool to rapidly evaluate the several hundred candidate interventions in early development,^10^ selecting the most promising candidates while also improving our critical understanding of viral pathogenesis and defining correlates of protection.

## 2 CONTROLLED HUMAN INFECTION MODEL AND HUMAN VIRAL CHALLENGE STUDIES

Human challenge studies have existed for over 200 years since Jenner and the development of the smallpox vaccine in 1793.^11^ In the modern era, the CHIM has become established for many pathogens in particular as the HVCM.^12^

Standard clinical trials carry a risk which must be balanced with the potential benefit. Likewise, an optimised risk‐benefit balance must be considered in all clinical studies. Despite the inherent risk of standard clinical trials, thousands of such studies are conducted successfully and safely each year, allowing the development of treatments and in some cases, the control or even eradication of diseases.

**Figure 1:**
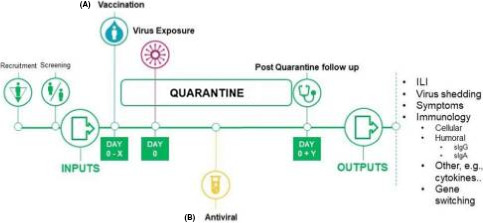
An outline of a HVCM study, specifically the Human viral challenge model. The study typically consists of inputs, such as the volunteers, their selection criteria, isolation in quarantine and exposure to a GMP virus. There are two treatment options; a vaccination/prophylaxis with an antiviral or b treatment with an antiviral. Outputs from the study summarised on the right, such as virus symptoms and virus shedding. *X* is the number of days before virus exposure vaccination may occur. *Y* is the number of days post‐virus exposure that a volunteer may be followed for^12^

HVCM studies are not dissimilar to standard clinical trials while adding another layer of complexity and safety considerations. The model (Figure 1) deliberately infects volunteers, with a challenge agent (CA) to determine the effect of the infection and the potential benefits of the experimental interventions. In standard clinical trials, it is extremely difficult if not impossible, to study the events occurring early during human infections. This is especially true in SARS‐CoV‐2 infections which are known to begin without recognisable symptoms. Therefore, HVCM studies can also be extremely valuable to understand better the pathogenicity of the virus before symptoms are apparent and the correlates of subsequent protection.

**Figure 2:**
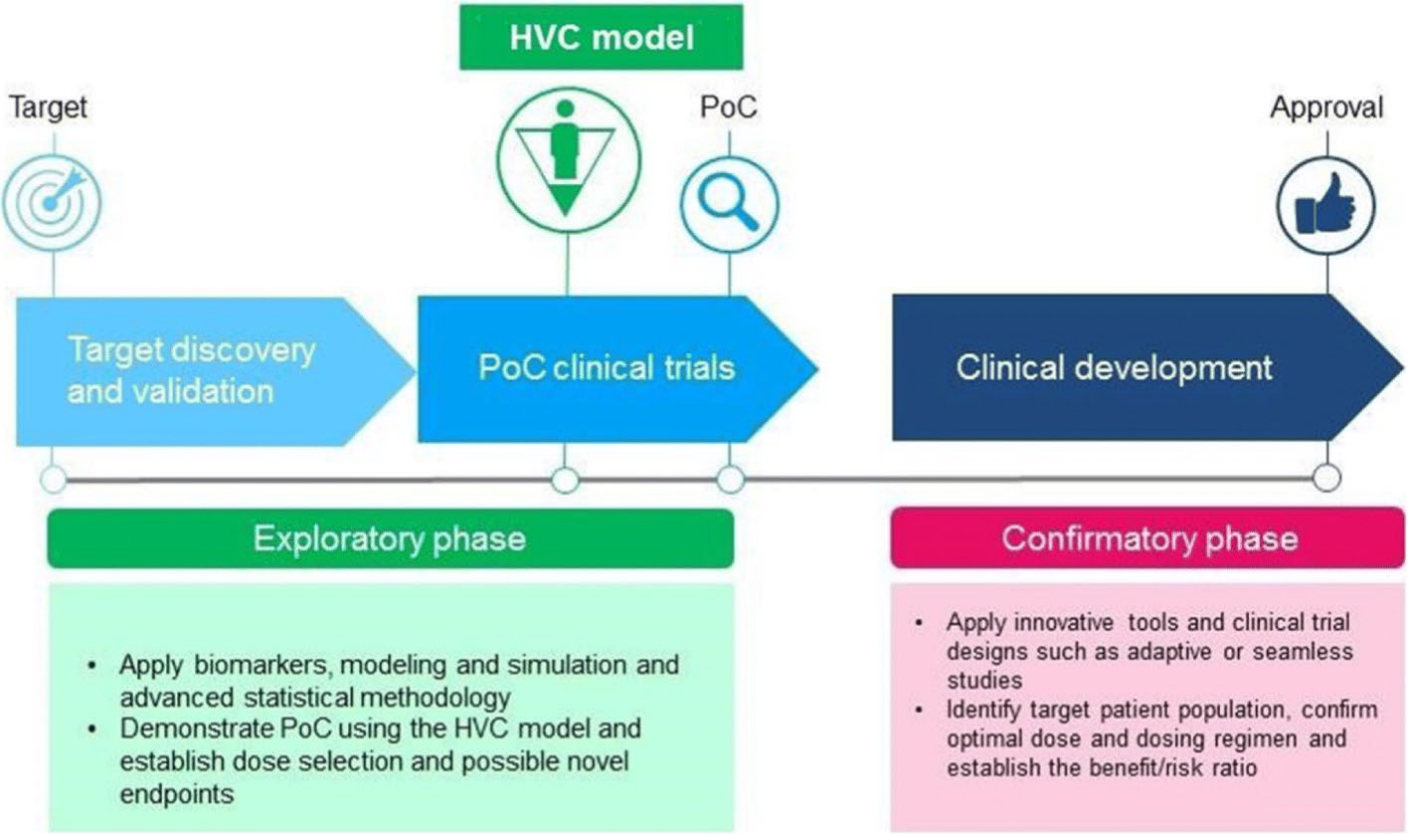
The role of the HVC model in the clinical development pathway. Short duration proof‐of‐concept studies, which incorporate the HVC model, typically include small numbers of subjects. The resulting safety and, particularly, efficacy data can more accurately guide decisions on whether to expose a larger number of subjects to promising candidate therapeutics in community‐based 14 field studies than conventional phase 1 safety data alone might otherwise^12^

HVCM studies can shorten the time to assess the efficacy of a new vaccine or treatment by combining the assessment of safety, with potential efficacy end points, as shown in Figure 2. HVCM studies may be used to accelerate the evaluation of vaccines and therapies against COVID‐19, leading to possibly earlier licensure.^13^ COVID‐19 is highly contagious and can cause severe illness and death. The understanding of the pathogenesis of this new virus is still developing, literally daily. Also, there is no standard therapy or vaccine available, although remdesivir has antiviral and clinical benefit even when started relatively late in the infection and has thus been given emergency use authorisation by the FDA.^14^

Previous successful human challenge studies have usually had a therapy available for treatment should a complication occur during an HVCM study, but this has not always been the case, as will be discussed later. A COVID‐19 HVCM study presents multiple ethics, safety, feasibility and regulatory hurdles.

However, HVCM studies, conducted ethically and safely, can have two significant benefits. Firstly, they can speed the development of vaccines and treatments, and secondly, possibly and more importantly, they can prioritise the most promising candidates amongst the hundreds currently being researched for more extensive studies.

On 6 May, the WHO issued its guidance on the conduct of COVID‐19 CHIM/HVCM studies, outlining “Key Criteria for the ethical acceptability of COVID‐19 human challenge studies”.^9^ The specified eight criteria are shown in Table 1.

**Table 1:** WHO criteria for the conduct of COVID‐19 HVCM studies^9^

Criteria	
1	There should be a strong scientific justification
2	The expected benefits should outweigh the risks
3	There should be engagement with the public, appropriate experts and policymakers
4	The research programs should be closely co‐ordinated between researchers, funders, policymakers and regulators
5	The study sites should allow the research to be conducted to the highest scientific, clinical and ethical standards
6	Participants selection criteria should limit and minimise risk
7	COVID‐19 challenge studies should be reviewed by specialised independent research ethics committees
8	There must be a rigorous informed consent procedure

Like all clinical studies, the criteria ensure that the optimised balance between risk and benefit is evaluated. A notable addition in the WHO guidance is criteria seven, “a specialised independent ethics committee” consisting of those with experience of COVID‐19 and HVC studies which is eminently sensible.

The choice of the challenge agent and the participants is particularly important concerning HVC studies where no effective treatment might exist. The authors describe their experience in addressing these two issues specifically, in the context of setting up the first pathogenic respiratory syncytial virus HVC study 10 years earlier, which has since been used to demonstrate the first human efficacy of several novel interventions, both therapeutic and vaccine.

The comparison of the previous development of a successful RSV HVC model and a possible future COVID‐19 HVC model is relevant given certain similarities, and the challenges faced with the two viruses as shown in Table 2.

**Table 2:** Relevant commonalities between RSV and SARS‐CoV‐2 virus/COVID‐19 and disease

Area	Commonalities
Genetics	RNA‐genome‐based respiratory viruses with broad genetic diversity
Host	Circulates in the human population as many distinct and evolving clades
Therapies	No vaccine or efficacious treatments is currently available
Pathology	Can cause serious lung disease and even death, mainly in certain population groups
Transmission	Early transmission from person to person is observed
Disease Risks	Some people are at high‐risk compared to others at lower risk of severe disease
Study volunteers	In healthy young adults, a population in which clinical studies would initially be conducted, infections tend to produce mild symptoms which may be difficult to identify making recruitment into community‐based field studies difficult
Adverse events	The potential for vaccine‐enhanced disease has been raised as a concern

## 3 THE CHALLENGE AGENT SELECTION AND SAFETY

Ensuring a safe and suitable CA is critical to ensuring an ethical and useful model. The pathogenicity of the CA must be such that the infection or disease produced is sufficient to generate suitable end points for the evaluation of relevant medical interventions while maintaining the clinical study volunteer's safety.

This balance has been successfully achieved in CHIM studies using viruses, bacteria and parasites, both attenuated and wild type in origin to successfully demonstrate vaccine and anti‐microbial studies successfully.

The CA may come from a regulated virus repository or a patient. It should be fully characterised and sequenced for mutations known to be related to severe disease, for example the D222G and D222N HA receptor‐binding site mutations for (H1N1) A/California/7/2009‐like (pdm09‐like) virus are related to poor prognosis would preclude isolates with these mutations as CAs.^15^

Animal models have in the past been often considered when developing a Good Manufacturing Practice (GMP) manufactured challenge virus stock for influenza,^16^ RSV^17^, ^18^ and HRV^19^, ^20^ to test the safety of the CA. But because of the recognised dissimilarity of viral pathogenesis in animals vs humans, such animal testing was not used to develop the RSV challenge agent. Animal models are part of the normal development pathway for human therapeutic agents, for both safety and efficacy testing. However, these models do have limitations; the transparent integration of efficacy and safety data derived from animal models and their translatability and predictive value for preclinical human studies can be limited. The predictive utility of such models are variable and complex, and they are inherently limited.^21^ In particular, animal models of respiratory disease are limited by the animals' physiology, immunology and the need for animal‐adapted pathogen strains in infection studies. Suitable animal models should be capable of generating data that are translatable to human studies, including pathogenicity and safety information. Animal models known to have a weak correlation with the human disease should be avoided. Famously, animal models of RSV infection, especially mouse models, have generally not been shown to be predictive of human infection and disease.

For COVID‐19 however, several animal models have been proposed, including ACE2 mice, ferrets, rabbits, non‐human primates and golden Syrian hamsters that may simulate human infection and disease.^22^ Whether or not they should be used to “safety test” a potential challenge agent (CA) should be debated.

## 4 PARTICIPANT SELECTION

Assuming that a suitable CA can be selected and a GMP lot produced, it is imperative to select a population with the lowest possible risks of developing severe complications of the infection.

As RSV can be a severe lower respiratory tract illness, in the authors' development of the RSV HVCM participating volunteers were carefully selected to be healthy males and females 18‐45 years of age and who had no history during adulthood of asthma of any aetiology or any use of a bronchodilator within the past year as well as other factors that may have complicated the analysis of the end points, for example hay fever or allergic rhinitis. Importantly, contact with people at risk of severe RSV infections, for example steroid use in the past month, chronic sinusitis and the presence of known immunosuppressive conditions, would also exclude a volunteer.

Similarly, the selection of volunteers would need careful consideration for a COVID‐19 HVCM. As an example, the age range would likely be limited to adults <30 years of age as the risk of complications increases in older age groups.^23^ The SARS‐CoV‐2 infection in younger age groups causes less severe disease, perhaps due to lower expression of ACE‐2 receptors in the respiratory tract.^24^ Multiple additional factors appear to place prospective participants volunteering at risk of serious COVID‐19 disease, including, but not limited to, race,^25^ BMI,^26^ sex^27^ and comorbidities, particularly in the elderly.^28^ The demographics of the participants must be carefully considered.

A small‐scale public consultation has been conducted amongst 20‐ to 40‐year‐old UK adults, focussing on the acceptability of a human‐controlled infection (CHIM) studies with wild‐type SARS‐CoV‐2 as a strategy to accelerate vaccine research. This group was consulted because they would likely comprise the enrolees of a putative CHIM because they have the lowest rate of severe manifestations of natural COVID‐19 disease in adults. Those consulted gave the opinion that a CHIM strategy would be acceptable even in the face of an uncertain risk: benefit ratio. A consistent view was that infection under highly controlled conditions in a hospital clinical research facility would be preferable to natural exposure and disease in the community. The necessary confinement during a CHIM would not be a bar to enrolment, subject to individual employment status.^29^

The availability of treatments with demonstrated efficacy in preventing or treating severe disease should it occur during a HVCM study is desirable. Still, as with COVID‐19, at the time the authors were establishing the RSV HVCM there was no effective treatment for adults, only a prophylactic agent for the use in small populations of high‐risk infants.^30^

As part of the design of a potential COVID‐19 HVCM study, if available an antiviral or other therapy, could be considered as a "shut down" switch. This would be triggered under certain circumstances, for example, viral load reaching a specific concentration threshold, or duration of viral replication, or a specific duration of early symptoms. Importantly, COVID‐19 HVCM studies must be conducted in facilities that are suitable and have access to emergency care facilities experienced with the care of severe COVID‐19 patients.

## 5 RELEVANT EXPERIENCE FROM THE ESTABLISHMENT OF THE RSV HVCM

Respiratory syncytial virus (RSV) is a paramyxovirus that infects more than 60% of children during the first year of life.^31^ This virus is associated with significant morbidity and mortality. RSV is particularly severe in immune naïve (infants),^32^ and amongst the frail elderly, RSV disease burden is similar to that of influenza.^33^ Globally, RSV infections are estimated to cause 66 000‐199 000 annual deaths in children under the age of 5 years and no vaccine or effective antiviral treatment for RSV disease exists.^34^ Passive monoclonal antibody prophylaxis (the only effective approved RSV intervention) is currently only applied to <5% of the at‐risk childhood population.^30^ Thus, there is a significant unmet medical need for effective therapies and preventions for RSV infection in the paediatric and high‐risk adult populations.

RSV‐directed drug efficacy is difficult to evaluate in healthy adult populations because natural RSV infections are mild to moderate producing symptoms generally difficult to distinguish from those of the common cold. Therefore, recruitment into community‐based field studies is difficult.

Animal models to evaluate novel antiviral drugs and vaccines against RSV correlate poorly to human infections and therefore, have limited value in the evaluation of potential human experimental interventions. The overreliance on animal models has held back the development of human interventions by well over a decade.^35^ The failed NIH RSV vaccine experience of the 1960s^36^ which resulted in the deaths of several immune naïve infant vaccine recipients after they subsequently acquired natural infection in the community in an uncontrolled manner further slowed the RSV vaccine development.

The authors set about developing a stock of virus that would safely and ethically mimic natural RSV infection and disease. A low‐passage stock of challenge agent from a clinical isolate was manufactured, and it was demonstrated to be able to generate reproducible data and be safe for use in healthy, carefully selected adult volunteers. The current lack of an effective RSV therapeutics underscored the reluctance to study experimental therapies directly in the vulnerable paediatric naturally infected population.^37^ Naturally infected immunocompromised adult population do not offer a suitable alternative, for a wide variety of reasons including that they are a small, geographically dispersed and difficult to recruit population.

These reasons have served to inhibit the preclinical and clinical development of RSV therapeutics. Numerous small molecules and novel therapeutics have been discovered with proven RSV antiviral activity in sub micromolar concentrations both in vitro and in vivo,^38^ but then failed to show an antiviral effect in humans due to many of these factors mentioned above.

To re‐evaluate the prevailing immune‐based model of RSV pathophysiology and to provide a practical means to conduct proof‐of‐concept RSV therapeutic trials, we sought to develop a safe, reproducible and well‐characterised human experimental RSV infection model in adult volunteers that paralleled natural RSV infection and disease.

The authors have already described the care that was taken in selecting participants for the RSV HVCM studies. To select a safe CA that was reliable for use in the RSV HVCM children with RSV were identified from a large regional paediatric hospital in Memphis, TN, USA. Between the years 2000 and 2005, aliquots of samples from 288 patients were obtained. The six aliquots with the highest viral loads in nasal washes (that were RSV‐A defined by both serotyping and genotyping) were taken into a manufacturing suite and processed following Current Good Manufacturing Practices (CGMPs).^39^

Samples were thawed and plaqued in FDA‐approved Vero cell cultures. Each of the six selected viruses produced visible cytopathic effects (CPE), and three individual plaques from each of the six viruses were selected and aliquoted. One aliquot from each of the 18 plaques was placed into a Vero cell serum‐free culture to assess quantitative viral growth kinetics. From these primary aliquots, cultures exhibiting the most optimal in vitro growth kinetics were then manufactured using GMP guidelines by passage in Vero cell culture roller bottles. One working stock that had been obtained from a single isolate (Memphis‐37) was then selected for final production. The CA was manufactured to a high titre due to the sensitivity of the virus to freeze/thaw cycles and that we were attempting to infect RSV immune experienced adults and therefore perhaps needed a high inoculum to infect.

After production, the fill‐finish was aliquoted in sterile glass vials and stored at −70 to −80°C. The final CA had been passaged only five times, thus limiting the number of mutations that could be introduced during manufacturing and hopefully ensuring the virus was typical of a wild‐type circulating strain. As with all live biologics administered to human volunteers in clinical studies, tests for purity and adventitious agents were extensive and followed FDA guidance documents for live viral vaccine production for human use.

The selection of the challenge agent is important; in this case, the patient from whom Memphis‐37 was originally isolated was a 4‐month‐old, 5.9 kg, African‐American male. He was previously healthy, and a full medical history was available for both mother and child, and repository consent was obtained. RSV exists as multiple different circulating strains of the virus; Memphis‐37 was shown to be broadly relevant to those currently circulating.

## 6 PRELIMINARY CLINICAL ASSESSMENT OF CHALLENGE AGENT

Thirty‐five volunteers pre‐selected to have low RSV Memphis‐37 neutralising titres were divided into five cohorts and in a sequential manner were inoculated with increasing quantities (3.0‐5.4 log PFU/person) of RSV Memphis‐37 intranasally.^40^ Each volunteer in a cohort received an aliquot of the same inoculum. Inoculation was by intranasal drops (0.5 mL/nare).

Between each cohort, the data were evaluated, including symptoms, signs and the viral load from daily nasal washes. After review by the safety committee, a decision was made as to whether or not to administer a higher inoculum of challenge agent to the next volunteer group. The lowest inoculum that reliably produced infection and symptoms was chosen.

**Figure 3:**
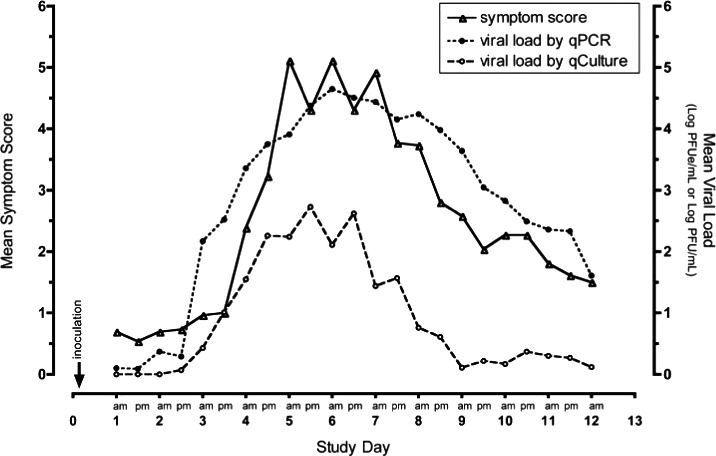
Viral load and disease over time in human volunteers. Timing of mean viral load and symptomatic disease. Mean data from all infected volunteers from each collection time point starting from Day 1 post‐inoculation are shown. The timing of peak viral load correlates with the occurrence of peak symptom severity. log PFUe/mL = log plaque‐forming unit equivalents per millilitre^40^

**Figure 4:**
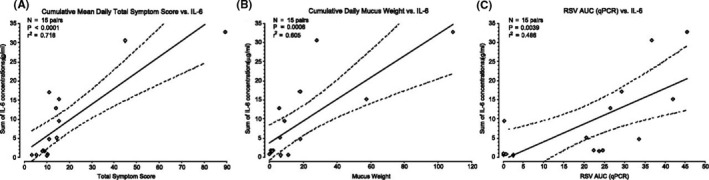
Relationships between IL‐6 concentration, disease severity, and quantity of respiratory syncytial virus (RSV). Concentrations of IL‐6 were measured in respiratory secretions of volunteers and were compared with disease measures and viral quantities. A, The cumulative sum of the IL‐6 concentrations vs disease severity as measured by individual volunteer cumulative symptom scores. B, The cumulative sum of IL‐6 concentrations vs disease severity as measured by individual volunteer cumulative nasal mucus weight. C, Comparison of the cumulative sum of IL‐6 concentrations vs area under the curve (AUC) viral load (quantitative real‐time reverse transcriptase‐polymerase chain reaction, qPCR). *P* values represent the probability that the slopes of the regression lines do not include a slope of zero. The dashed curved lines indicate the 95% confidence interval of the slopes of the regression line (solid line). Similar statistically significant direct relationships were observed when viral AUC was measured by quantitative culture^40^

Overall, 77% of volunteers consistently shed the virus. Infection rate, viral loads, disease severity and safety were similar between cohorts and appeared unrelated to the quantity of RSV received. Symptoms began near the time of initial viral detection, peaked in severity near when viral load peaked and subsided as viral loads (measured by RT‐PCR) slowly declined as shown in Figure 3. Also, viral loads correlated significantly with intranasal proinflammatory cytokine concentrations, notably IL‐16, as is also seen with COVID‐19.^41^, ^42^ Figure 4 Increased viral load correlated consistently with increases in other multiple different disease measurements (symptoms, physical examination, amount of nasal mucus). In this safety study, the viral load appeared to drive disease manifestations in humans with RSV infection, thus helping to understand RSV human disease pathogenesis.^43^ No safety issues were observed in this study. Mimicking natural RSV infection and disease makes this model useful in providing early proof of concept of various treatments, therapies and vaccines.

An editorial in the American Journal of Respiratory and Critical Care Medicine, described the utility of the RSV HVCM saying; “This model permits the relatively quick and efficient study of new therapeutics in humans and assists in making critical decisions whether to advance a product into costly human trials in populations at highest risk of disease; children, elderly or immunocompromised patients. This constitutes a major and welcome advance in the field of RSV.”^44^

In conclusion, a safe and reproducible human experimental model of RSV infection and disease was developed, and the developmental process may parallel potential COVID‐19 HVCM development in several ways. The RSV HVCM has already proven its worth in quickly establishing important human therapeutic efficacy and in speeding the development of experimental interventions which are now being conducted in high‐risk populations.^45^ Additionally, the RSV HVCM has provided the first evidence of RSV vaccine efficacy in producing sterilising immunity and disease severity reductions.^46^

## 7 DEVELOPING A COVID‐19 HVCM

As outlined above, an HVCM can be developed, even when safety and ethical issues may be present as long as each is addressed in a carefully thought out and thoroughly interrogated manner. If a COVID‐19 HVCM is developed, it would be of enormous value.

There are challenges that specifically face the establishment of a COVID‐19 HVCM. Some of these challenges are truly unique while most have been, to various degrees, faced in the development of other viral challenge models. Unique aspects include the newness of the virus and the extreme public awareness of it. This means that large amounts of new information arriving even on a daily basis need to be evaluated and incorporated into trial design. Amongst others, this new information might affect strain selection of the challenge agent, human severity risk factors affecting subject selection, and the use and timing of viral shut‐off therapeutics incorporated into the HVCM. Protocols thus need to be rapidly adaptable to such potential changes, and any necessary revisions must be able to be quickly implemented. Similarly, the model is likely to generate valuable new clinically and scientifically relevant data at speed and it will be the duty of the investigators to ensure it is shared with colleagues in an efficient and open manner.

COVID‐19 HVCM challenges which have been faced in previous HVCMs include protection against escape from the facility, safety of staff, handling of long‐term shedders and advanced preparations in case volunteers become severely ill. The widespread community presence of SARS‐CoV‐2 infections worldwide reduces the concern of the virus escaping from the facility, but adds to the importance of selecting a currently circulating strain of virus for the challenge agent. The facilities themselves are important in any HVCM study as the days of wooden huts on Salisbury Plain during the 1950‐1990s are behind us. Current HVCM facilities are still too‐often retrofitted phase I units or converted hospital wards or buildings. Purpose‐built facilities are preferable and should be able to contain a pathogen within an enclosed environment with volunteers remaining in single, negative pressure, HEPA filtered, externally vented rooms. Such facilities have been used in some, but not all previous respiratory virus CMs. For COVID‐19 HVCM, consideration might also be for waste water systems to be separated from the main systems of the building. As currently practised in COVID‐19 hospital wards, appropriate personal protective equipment and training is a necessity and regular staff testing for virus should be considered, given the virus’ ability to cause asymptomatic and pre‐symptomatic shedding.

Interestingly, in the experience of both authors, the desire of volunteers to leave once admitted to isolation is rare, and usually caused by an unexpected social/family event, rather than boredom. Indeed, the team caring for the volunteers ensures they are “occupied” and the provided social media access means they rarely feel alone. However, if a volunteer needed to leave the facility consideration would be given to isolation at home, available antiviral treatments and appropriate support and home monitoring as has been accomplished in other HVCMs.

There is an important difference between long‐term PCR positivity, and long‐term shedding of infectious viral particles,^47^ and risks of viral transmission to others. Occasionally, volunteers during other respiratory challenge studies have been asked to remain in the facility if their shedding is prolonged. As practised in community‐identified SARS‐CoV‐2 infections, aspects of home quarantine, with confirmed repeated PCR negativity, would likely be incorporated into the HVCM.

As in all HVCMs, there is a small risk that a volunteer could develop a serious complication. It is therefore essential that a close relationship is established and maintained with a nearby hospital with suitable intensive care services, bed space and experience in treating severe COVID‐19 disease. Emergency facilities and medical systems on‐site should be capable of treating COVID‐19 more minor complications, and the on‐site medical staff should be carefully selected with experience in treating the disease. Such systems have been part of the HVCMs in which these authors have been involved (Figure 4).

Development of a safe and successful model could establish early human proof‐of‐concept and accelerate phase 2 and 3 design and development. A COVID‐19 HVCM could also prioritise/triage the community‐based field studies of therapeutic and vaccine candidates, ensuring that the most promising are prioritised for assessment during subsequent waves of infection where the recruitment pool of potential research subjects being naturally infected may still be limited due to social distancing or between waves of disease.

There are potential safety advantages of a COVID‐19 HVCM over traditional vaccine community‐based field studies. Firstly, for vaccine efficacy evaluations, a sufficient number of subjects need to become infected. In community‐based field studies, to ensure enough infections occur, a large number of subjects must be vaccinated and then followed up. With an HVCM, a much smaller number of volunteers would be subject to the potential initial safety risk of the experimental vaccine. Secondly, the safest place for a vaccine and its interaction with the virus to be evaluated would be in a controlled medical setting such as that provided in a HVCM unit with established access to the full range of immediate medical care, this is particularly true for a vaccine where the risk of vaccine‐enhanced COVID‐19 disease^48^ is a possibility.

There is also recent precedent for vaccine licensure based on data from HVCM studies. Examples include cholera and typhoid vaccines.^49^, ^50^, ^51^ The use of the COVID‐19 HVCM may enable emergency use authorisation/licensure far more rapidly than a conventional approach, without replacing it.

## 8 CONCLUSION AND MOVING FORWARD

We have demonstrated in the past that the development of a new HVCM can be achieved despite formidable obstacles. Importantly, we have used the RSV HVCM successfully in multiple studies and have demonstrated that such HVCM studies speed clinical development of experimental interventions, including vaccines. We propose that due to the time required to identify and manufacture a CA, the discussion must start immediately.

Also, the cost and time of developing such a model are not directly proportional. A wide range of isolates may need to be collected from around the globe, given the mutability of this RNA virus already observed and the developing phylogenetic network.^52^ Testing will take time before a suitable isolate can be found. A bank of seed viruses may need to be developed and characterised, followed possibly by multiple GMP batches of viruses. This time should be utilised to work through the ethical, safety and practical concerns of conducting the first COVID‐19 HVCM safely.

Working groups should be established, including experts with direct experience of controlled human infection studies, the regulatory agencies, appropriate biomedical research ethics committees and institutional review boards. A critical point for discussion should be how similar to wild type the chosen challenge virus should be, allowing for safety and manufacturing considerations, such that the data generated from the model is applicable to naturally occurring infections. Ten years ago, we developed an RSV HVCM that is now frequently used, and believe that a similar properly designed and orchestrated safe and reproducible COVID‐19 HVCM is possible and would be of substantial global benefit.

## CONFLICT OF INTEREST

This is a review article, and all various declarations have been made on the relevant papers by the authors and cited amongst the references. However, the authors have conducted CHIM/HVCM studies and therefore declare the following for clarity. Robert Lambkin‐Williams is an Independent Consultant Virologist with the consulting company VirologyConsult Ltd. UK. He previously worked at Retroscreen Virology Ltd, which was renamed hVIVO Services Limited. This company was a Contract Research Organisation specialised in respiratory viruses and in particular the human viral challenge model. He still owns some shares in this company and does provide some consulting services to them. He provides consulting services in the area virology to other companies as an independent advisor. John DeVincenzo received RSV‐related research contracts (through the University of Tennessee) and consultancy fees from Janssen, Reviral, Pulmocide, ADMA Biologics, Ark, Pfizer and MedImmune/AstraZeneca, and has received consultancy fees from VirBio and Enanta.

## AUTHOR CONTRIBUTIONS

Robert Lambkin‐Williams: Writing‐review & editing (equal). John DeVincenzo: Writing‐review & editing (equal).

### Peer Review

The peer review history for this article is available at https://publons.com/publon/10.1111/irv.12797.

## Data Availability

This manuscript is a commentary piece and any raw data; therefore, this section is not applicable.
